# Development of the IRIS-AR strategy: an intervention to improve rates of accrual and retention for the VTE-PRO randomized controlled trial

**DOI:** 10.1186/s13063-019-3536-8

**Published:** 2019-07-19

**Authors:** Christine Fahim, Danielle Hylton, Marko Simunovic, John Agzarian, Christian Finley, Wael C. Hanna, Yaron Shargall

**Affiliations:** 1 Bloomberg School of Public Health, Johns Hopkins University, Hampton House, Room 663, 624 N Broadway, Baltimore, MD 21205 USA; 2 Department of Surgery, McMaster University, Hamilton, ON Canada

**Keywords:** Accrual, Retention, Theoretical domains framework, Behavior change wheel, Randomized trials

## Abstract

**Background:**

The Venous Thromboembolism Prophylaxis (VTE-PRO) randomized trial is a pilot study evaluating the impact of extended-duration prophylaxis on venous thromboembolic events in patients undergoing lung cancer resection. Enrolled VTE-PRO participants self-inject either low-molecular weight heparin or a saline placebo for 30 days postoperatively. Study outcomes include feasibility, incidence of venous thromboembolism, and venous thromboembolism-related morbidity and mortality. Initial analyses demonstrated low rates of accrual and retention for the VTE-PRO pilot. Therefore, the purpose of the current study was to develop a knowledge translation intervention to improve VTE-PRO pilot trial accrual and retention.

**Methods:**

Eligible participants were surveyed to identify the barriers to VTE-PRO participation. The Theoretical Domains Framework was used to categorize these barriers. Barriers were mapped to the capabilities, opportunities, and behavior (COM-B) behavioral change wheel to identify potential interventions to support trial accrual and retention. The resulting knowledge translation intervention was titled Inform, Remind, Involve and Support to improve Accrual and Retention (IRIS-AR). Key informant interviews with patients were held to refine and confirm the validity of identified barriers and perceived acceptability of the proposed IRIS-AR intervention. Institutional Review Board approval was granted for this study.

**Results:**

The resulting intervention included: information booklets and counseling sessions to identify unique participant challenges to trial participation (Inform); daily reminders to administer injections (Remind); involvement of family/caregivers in study processes (Involve); and leverage of an existing home-care nursing program to provide injection support when needed (Support). Twenty-six key informant participants were interviewed. The most common barriers to trial participation included lack of social support and fear of needle injection. Participants generally supported use of information booklets, involvement of family/caregivers, and support by a home-care nursing program; however, not all supported the use of daily reminders.

**Conclusion:**

Developed using theory and integrated knowledge translation, the IRIS-AR presents a patient-centered intervention that leverages existing programs to promote trial engagement. The proposed strategy can likely be adapted to improve compliance with other patient-directed interventions.

**Trial registration:**

ClinicalTrials.gov, NCT02334007. Registered on 8 January 2015.

## Background

Despite the recognized importance of randomized controlled trials (RCTs) as the cornerstone of evidence-based medicine, RCT accrual and retention remain significant challenges for researchers. Over the past three decades, researchers have identified a number of barriers to RCT accrual and retention, including caregiver preferences to not participate, unease regarding randomization, limited understanding of patients regarding trial goals/risks, and unwillingness to comply with study demands [1–5]. Other pertinent reasons reported in the literature include symptom burden, altered mental status at time of consent, and fear of adverse events [6].

To date, solutions to overcome RCT accrual and retention barriers include selection of a motivated target population, the introduction of a run-in period to exclude noncompliers, employing the use of a flexible intervention regimen, shortening the rate of study follow-up, and avoiding outcomes that lead to large amounts of missing data [7–10]. However, systematic reviews suggest that the effectiveness of such strategies remain in question [4]. Moreover, most of these solutions are not applicable to studies with short follow-up periods, such as surgical trials [4, 8].

It is therefore necessary to identify a pragmatic approach to improve RCT accrual and retention rates, particularly for trials that involve a patient-led intervention component (e.g., self-administered injection). Knowledge translation (KT) is the dynamic process of synthesizing, disseminating, exchanging and applying evidence to practice. KT experts suggest the use of theory in intervention design to comprehensively identify the behavioural mediators that influence a behaviour of interest [11–13]. For instance, hospital administrators aiming to improve the practice of staff hand washing must first determine the individual, group, or environmental factors that reduce hand washing compliance. Moreover, failure to involve the target population in the process of intervention development can lead to inappropriate or unnecessary interventions that are less likely to be effective [14, 15].

We used principles of KT, particularly the use of theoretical frameworks, to design the IRIS-AR (Inform, Remind, Involve and Support to improve Accrual and Retention), a support strategy aimed at improving accrual and retention for an ongoing randomized trial involving the use of self-directed heparin injections in a postoperative thoracic population. In this report, we describe the methodology used to develop the IRIS-AR support strategy and the findings of key informant interviews conducted with participants in the postoperative thoracic population to refine and confirm the acceptability of the proposed intervention components.

## Methods

### Study setting

This study took place at St. Joseph’s Healthcare Hamilton (SJHH), a leading tertiary academic cancer center in Ontario, Canada, that serves a population of 1.7 million people. The IRIS-AR trial was conducted as a supplement study to improve rates of accrual and retention for the Venous Thromboembolism Prophylaxis (VTE-PRO) randomized trial [16]. Venous thromboembolism (VTE), which includes both deep vein thrombosis and pulmonary embolism, is a common postoperative complication that affects up to 15% of patients undergoing lung resection for malignancy [17, 18]. Patients who experience VTE events following lung resection have a mortality risk of up to 14.3% compared to less than 2% for patients who do not experience a VTE event [19]. The VTE-PRO trial is a pilot study that compares in-hospital prophylaxis to extended-duration, 30-day, postdischarge prophylaxis for patients undergoing lung cancer resection [16, 19]. In the VTE-PRO trial, participants are required to self-inject either low-molecular weight heparin (intervention arm) or a saline placebo (control arm) once daily for 30 days after surgery (primary endpoint), following hospital discharge. Primary outcomes included feasibility and safety with VTE incidence. Secondary outcomes included 90-day survival.

To date, most thoracic surgery trials at our center involved the use of in-hospital interventions or interventions that were ordered or delivered at the time of routine clinical visits; once patients consented, their participation in a given trial was relatively straightforward. In contrast, VTE-PRO trial patients must actively engage in the study intervention away from the constant support of the study team members (daily, at-home self-injections for 30 days following postoperative discharge from hospital). Compared to previous studies, our study team observed lower rates of accrual and retention for the VTE-PRO pilot which threatened the validity and generalizability of the resulting study findings. Therefore, the impetus to develop IRIS-AR was to overcome low rates of accrual and retention to improve the likelihood of trial feasibility and success prior to implementation of the full-scale, multicenter VTE-PRO study at our national and international partner sites.

### Study design

We conducted a theoretically rooted qualitative study, meaning we used a theoretically rooted framework to inform the design and analysis of our study. As part of routine monitoring of the VTE-PRO pilot, study coordinators prospectively surveyed in detail all eligible VTE-PRO trial participants, including those who chose not to participate or dropped out of the VTE-PRO trial, to determine reasons for nonparticipation or drop-out. The following factors were identified as the top barriers to trial participation: fear/changed mind/feeling overwhelmed (38%); inability to comply with injection (10%); and family/gatekeeper influence to withdraw participant (10%). Many participants felt that it was “too much” or “overwhelming” to administer the daily injection, while transitioning to usual life following their thoracic surgery. Family members who influenced participants to withdraw were typically concerned with issues surrounding randomization, administration of the injection, and fear of adverse events.

We mapped these data to a framework, the theoretical domains framework (TDF), to identify underlying theoretical mediators that influenced participant decisions to not participate in, or withdraw from, the VTE-PRO trial [11, 12]. Once these theoretical domains were identified, they were mapped to a framework (the capabilities, opportunities and behavior (COM-B) behavior change wheel) that matches theoretical mediators with corresponding interventions [13]. Corresponding COM-B interventions were selected to develop the IRIS-AR intervention components.

**Fig. 1:**
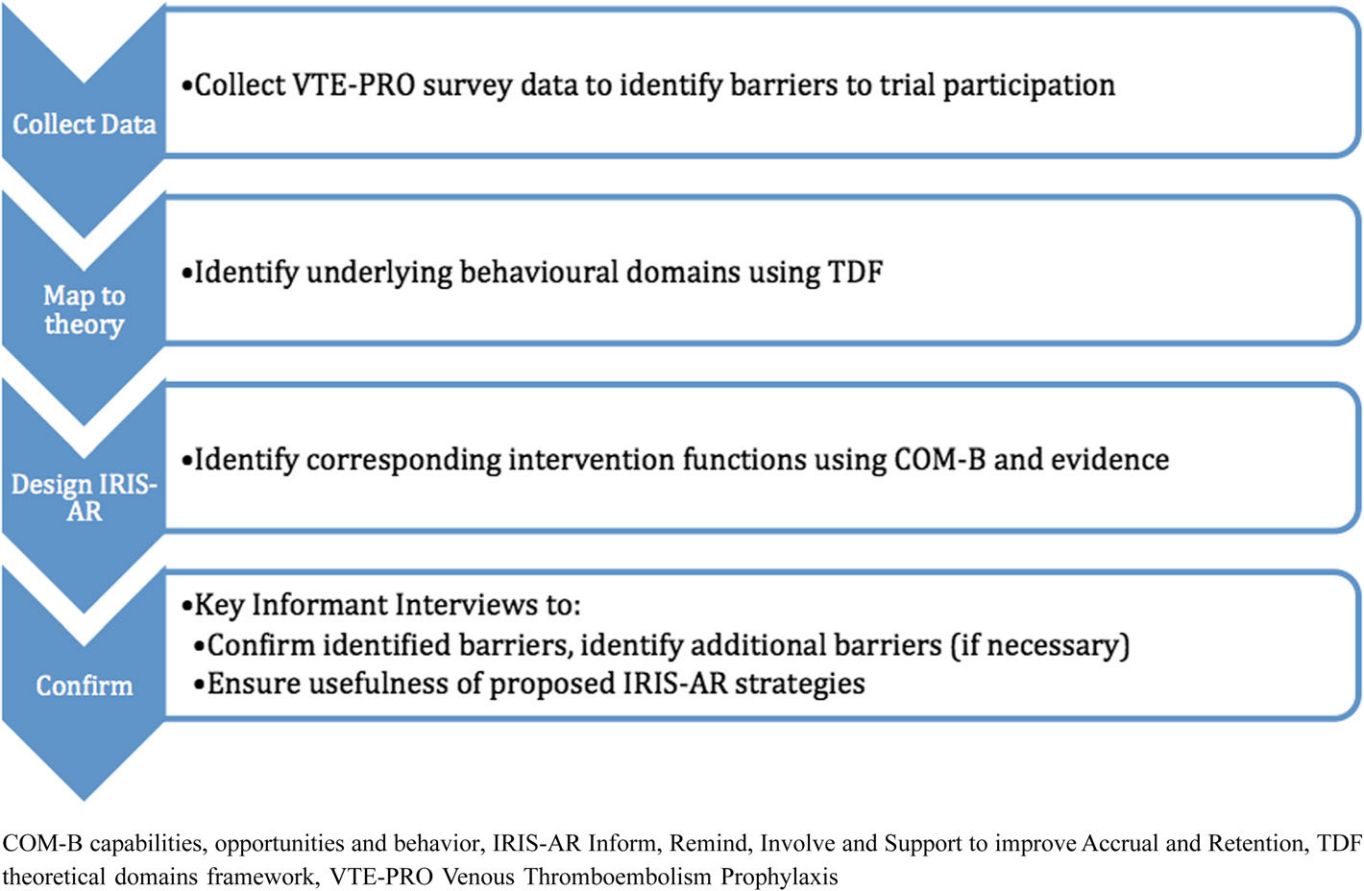
Overview of the study design

Following IRIS-AR development, key informant interviews with VTE-PRO trial participants were conducted. The purpose of the interviews were to: 1) confirm or refine identified barriers to accrual and retention, as identified using the VTE-PRO prospective survey data; 2) identify additional barriers not identified in the survey data, if relevant; and 3) assess the perceived usefulness and acceptability of the proposed IRIS-AR support strategy. A visual overview of the study design is provided in Fig. 1.

Institutional Review Board approval was received by the Hamilton Integrated Review Ethics Board (HiREB).

### TDF and COM-B behavior change wheel

KT experts recommend the use of theory to develop behavioural interventions [20]. However, there are many theories that can be used to inform the design of an intervention, which makes it difficult to justify the use of one theory over another. In response, meta-frameworks can be used, such as the TDF, which integrates over 30 psychological theories into a single framework comprised of 14 behavioural domains [11, 12]. By using the TDF, researchers need not prioritize the use of one theoretical framework over others. Rather, the TDF allows for a systematic identification of the motivational, social, physical and environmental factors that can influence behaviour.

KT literature suggests that the use of theoretically rooted interviewing elicits more findings on mediators to behaviour change, compared with traditional interviewing techniques that may overlook potential barriers or facilitators [21]. We created an interview guide rooted in the TDF. The 14 domains of the TDF include: knowledge; skills; social/professional role and identity; beliefs about capabilities; optimism; beliefs about consequences; reinforcement; intentions; goals; memory, attention and decision processes; environmental context and resources; social influences; emotion; and behavioral regulation [12]. While the TDF was initially created to identify the factors that influence health professional behaviour, the framework has since been extended to other populations and contexts, including patient populations.

Identified theoretical domains can be directly mapped to the COM-B behaviour change wheel to identify corresponding strategies to mitigate the desired behaviour [13]. The COM-B outlines six sources of behaviour; these include: social and physical opportunity; automatic or reflexive motivation; and physical or professional capability*.* Corresponding to these behavioural sources are nine intervention functions that can be used to influence the corresponding behavior of interest [13]. The intervention functions include: education; persuasion; incentivization; coercion; training; enablement; modeling; environmental restructuring; and restrictions [13]. Used together, the TDF and COM-B allow for a systematic analysis of barriers and facilitators impacting behavior and prioritization of potentially relevant intervention functions (i.e., guidance on which of the nine interventions on the behavior change wheel should be selected). Implementation strategies (i.e., the specific activities that will be used to execute the intervention functions) can then be identified and tailored to the target population. This process is in contrast to the more commonly employed method of implementing ‘common-sense’ solutions, which often do not result in the desired behavior change [14].

### IRIS-AR design

Barriers to VTE-PRO accrual and retention were mapped to the TDF to identify underlying behavioral constructs. These constructs were then mapped to the COM-B to identify corresponding intervention functions. For example, survey data identified ‘fear of injections’ as a theme to study nonparticipation. This theme falls into the theoretical domains of ‘skills’ and ‘beliefs about capabilities’ (e.g., fear of incorrectly administering the needle) and ‘emotion’ (e.g., fear of needle pain; anxiety regarding self-administration of injection). These theoretical domains correspond with the COM-B behavioral components of ‘capabilities’ and ‘motivation’. Using the behavior change wheel, we believed the interventions of ‘training’, ‘enabling’, and ‘educating’ patients to administer injections and overcome fears would be effective. This mapping approach was used to design the IRIS-AR.

### Data collection

A semistructured interview guide was designed to confirm and refine identified barriers to VTE-PRO accrual and retention. This process also ensured that any pertinent themes not identified in the VTE-PRO survey data were subsequently captured in the key informant interviews. The interview guide included questions relevant to each of the TDF domains and was modeled after similar examples in the KT literature [22–24]. Participants were also asked to comment on the perceived acceptability of the proposed IRIS-AR intervention components.

### Study participants

Key informant participants were purposefully recruited to ensure representation of participants who had completed, or were in the process of completing, the VTE-PRO trial intervention (30-day intervention period), participants who dropped out of the trial, and those who never consented to participate in the trial. Recommended sample sizes for qualitative research vary significantly from 6 to 30 interviews [25–27]. Researchers recommend continuing with key informant interviews until data saturation is reached, or until no new themes appear in the data. Francis et al. provide a guide for sample size estimations for theoretically rooted interviews, which sets a minimum sample size of *n* = 10 interviews, followed by an additional three interviews for which no new themes arise [28]. Therefore, we anticipated that a minimum of 13 interviews would be conducted, but continued interviews until data saturation was reached.

### Data collection and analysis

Two researchers trained in qualitative methodology telephoned potential participants to invite them to participate in the key informant interviews. All recruited participants consented to be approached for an interview. Following verbal consent, all data were recorded and transcribed, with identifiers removed. These de-identified data were analyzed for emergent themes using thematic analysis. The purpose of thematic analysis is to identify and categorize overarching, emergent themes present in the data. Two researchers (CF and DH) conducted the interviews and double-coded the interview data. The TDF was used as the initial coding framework and was compared with the findings from the survey data. A second round of coding using thematic analysis was then conducted. Any discrepancies in coding were resolved through a consensus process.

### Researcher characteristics and reflexivity

The characteristics of SJHH are described in the study setting. Interviews were conducted by nonclinician researchers not directly involved in the VTE-PRO randomized trial to minimize potential bias. The qualitative analysis team members (CF and DH) are academic researchers with nonclinical backgrounds. The willingness of participants to comment on their experiences with the VTE-PRO trial may have been impacted by knowledge (or lack of knowledge) of the researchers’ backgrounds. Study interviews were conducted by telephone at the convenience of the study participant. While an interview script was used to guide the interviews, we used a semistructured approach to allow participants to speak freely and have increased control over the interview process. CF, the qualitative lead, is an expert in qualitative research and the use of theoretically rooted interviewing for implementation science research.

## Results

Identified theoretical domains that influenced trial participation and compliance included: knowledge; beliefs about capabilities; beliefs about consequences; intentions; memory, attention, and decision processes; social influences; and emotion*.* Table 1 provides a detailed description of identified TDF domains and corresponding COM-B interventions.

**Table 1:** Identified theoretical domains pertaining to VTE-PRO trial participation and compliance

Barrier/facilitator to VTE-PRO trial	TDF domain	TDF construct group	COM-B component and intervention function	IRIS component
B: Misunderstanding of study processes (i.e., coming to hospital for injections) F: Understand and are comfortable with study processes	Knowledge	Knowledge of task environment	Psychological capability – environmental restructuring/modeling Physical capability – education	Inform
B: Fear of inability to administer heparin injection F: No fear of injection	Beliefs about capabilities	Perceived competence	Reflective motivation – training	Inform, support
B: No direct benefits to self by participating B: Fear of complications F: Participation will further science, help others and themselves	Beliefs about consequences	Beliefs Anticipated regrets Outcome expectancies	Reflective motivation – training	Inform
B: Forget to self-administer injection daily F: Remember to self-administer injection daily	Memory, attention and decision processes	Attention control Memory	Psychological capability – modeling; environmental restructuring	Remind
F: Family/friend concerns about trial participation are considered, yet ultimately patients make final decision regarding participation F: Social networks facilitate motivation, support, reminders for needle injections, and assistance with injections	Social influences	Social pressure Social support	Social opportunity – education; persuasion	Involve
B: Feelings of fear, anxiety or stress pertaining to upcoming surgery	Emotion	Fear Anxiety Stress Depression	Automatic motivation – enablement	Inform, involve, support

*B* barrier, *COM-B* capabilities, opportunities and behavior, *F* facilitator, *IRIS* Inform, Remind, Involve and Support, *TDF* theoretical domains framework, *VTE-PRO* Venous Thromboembolism Prophylaxis

Table 2 outlines the initial IRIS-AR intervention components and corresponding evidence. The first iteration of IRIS-AR was comprised of four intervention components. The first component (‘inform’) ensures participants are informed of the study purpose and processes, and that all concerns regarding trial participation are addressed. The second component (‘remind’) provides participants with daily reminders to administer their injections. The third component (‘involve’) involves caregivers and family members in the study process to alleviate study concerns and ensure that the participant is supported throughout the study period. The final component of IRIS-AR (‘support’) offers nursing support for patients unable to self-administer the heparin injection. The ‘support’ component leverages the SJHH Integrated Comprehensive Care (ICC) program, which is a novel home-care program consisting of nurses, physiotherapists, respiratory and occupational therapists, and dieticians [29]. A nurse coordinator actively follows every postoperative thoracic patient, and is involved in in-hospital patient assessment, development of a discharge plan, and coordination of the outpatient ICC care team [29]. All patients are contacted within 24 h of discharge, and personalized care is provided to each patient. The home-care ICC team was made available to patients who required further assistance with their needle injections.

**Table 2:** Description of first iteration of the IRIS-AR support strategy, prior to key informant interviews

IRIS intervention component	Description of intervention	Operationalization of intervention strategy
Inform	A counseling session and information booklets to ensure participants are sufficiently informed on: • the risk of venothrombotic events; risks of study participation • the study purpose, intervention and participant expectations • common concerns/questions pertaining to VTE-PRO trial processes	Eligible participants will meet with a study coordinator who will thoroughly explain the VTE-PRO trial and counsel study patients or their caregivers. In this context, counseling refers to identifying and addressing specific patient concerns regarding the study. Counseling fosters intrinsic motivation and addresses patient concerns otherwise unknown to the study team. A standardized discussion template will be used to make notes specific to each patient. This will aid the research team in identifying supports that are acceptable to the individual patient. The counseling session will take place in-hospital, for approximately 20 min, following initial consent. Eligible participants will also be provided with a standardized information booklet that will address common concerns pertaining to injections, fear of randomization, and study processes. The booklet will also contain testimonials by previous participants regarding their experiences and recommendations on how to remain compliant with the daily injections. Participants will have the option to utilize any of the components provided in the remaining IRIS-AR components (‘remind’, ‘involve’, ‘support’)*,* as per their individual needs
Remind	Daily or weekly tailored reminders via text message, telephone, and/or email (as per patient preference).	Study coordinators will send messages tailored to the concerns, needs and milestones of each individual patient. If employed actively and creatively, these prompts may demonstrate a significant impact on study retention, at a relatively low cost
Involve	Following participant consent, caregivers and/or family members/friends will be invited to participate in the ‘inform’ session.	Caregivers/family members/friends will participate in the ‘inform’ session to understand all study processes, participant responsibilities, and will be afforded the opportunity to ask questions to the study coordinator Caregivers/family members/friends will be encouraged to remind the participant to administer the daily injection Caregivers/family members/friends may be trained to administer the injection to the participant, in the event that the participate is unable/fearful and/or does not wish to use the ICC nursing support
Support	Following participant consent, the at-home team of the ICC program will travel to the participants’ home to administer the daily injection, for participants unable to administer/fearful of administering injection	A nurse will travel to the participant’s home to administer the needle. The nurse will work with the patient to overcome fears of the needle and will encourage the patient to self-administer the injection

*ICC* Integrated Comprehensive Care, *IRIS-AR* Inform, Remind, Involve and Support to improve Accrual and Retention, *VTE-PRO* Venous Thromboembolism Prophylaxis

Following the development of the first IRIS-AR iteration, interviews with key informants were conducted. Data saturation was reached at 26 interviews. Participants confirmed the validity of barriers and facilitators that were identified via the survey data, and provided additional insights into the barriers and facilitators impacting VTE-PRO trial participation (Table 3).

**Table 3:** Key informant interview themes

	Facilitator to VTE-PRO trial participation	Challenge to VTE-PRO trial participation
Knowledge of study procedures/objectives	“I was under the impression that [the study pertained to] blood clots because I was going in for surgery and had a prior history of blood clots … so it was basically no big deal” (P9) “I recall the self-injection, and some would be placebo and some would be the real thing [heparin].” (P12) “I thought it was pretty clear” (P17)	“At one point I thought that the study was 3 months long and that really gave me a little bit of anxiety and I was going to quit, but once I found out the actual time reference (30 days) I had no problem continuing” (P8) “I thought a nurse would be coming out [to my home] to do [the injection] for me, and they said no that’s not the way it works. I was waiting for the nurse to come. I didn’t realize it was going to entail self-injections” (P10) “I don’t drive, I don’t have a car and I am 88 years old, and I just couldn’t be bothered to [go back and forth to hospital]” Interviewer: So was it your impression that you had to go to hospital to receive the injection for 30 days? “Yes” (P11)
Support from family/friends	“My wife agreed with me that it [VTE-PRO participation] was probably a good idea she gave me a couple of injections … my wife was 100% involved … I think it was quite important that there is somebody there for the person” (P3) “My husband is always supporting me in everything … but yeah, maybe other people might want more people involved”	“It might irritate some people [if family was involved], and they might think oh yeah that was a good idea if I had my family and my friends to remind me, and then get a phone call and be overwhelmed.” (P6) “I don’t have very supportive friends. They were like ‘what, you signed up for this?’ and I was like, ‘yeah, it’s all volunteer’.” “The time I didn’t do my injection, it wasn’t that I forgot, I think it was kind of laziness on my part. You know what I mean, like to get out of bed and actually do this … and I said I am going to stay right here in bed” (P7)
Reminders to take injection	“[My wife and I] reminded each other really. It was basically around 12 noon every day that we did the injection” (P13) “My family was supportive, my wife was an alarm clock [reminder]”	“There were two successive days that for some reason I forgot [to take the injection]” (P17) “A couple times if my husband hadn’t asked me if I had done it [the injection] then I would have forgotten to do it. There was one day that I completely forgot, and I didn’t remember until the next day” (P8)
Ability to self-administer injection	“It was not hard or anything, it was a small needle and you just stick it in your stomach and it was all good” ((P2) “They are not big needles and they are not a big deal and they don’t go deep … it”	“I had my granddaughter coming over at seven in the morning to get the injection to me and then … a week and a half later I started doing them myself” (P7) “I am worried I am going to fill the syringe incorrectly … you know pulling the plunger out and getting the air out … I’m not going to self-inflict this worry” (P10) “A lot of people are afraid of needles … trying to get them to give one to themselves would be hard” (P9)

*VTE-PRO* Venous Thromboembolism Prophylaxis

### Facilitators to study accrual

Participants who completed the VTE-PRO randomized trial cited consistent reasons for trial participation including an intrinsic desire to participate in research, help future patients with similar conditions, and potentially improve their outcomes, as seen in the following quotes:

“It’s just to help others … and basically, give you a chance to see what will work and what won’t work” (participant (P6)

“I already had [surgery] three other times and fortunately I had not had any blood clots, so I thought that I would do my part and help the accumulation of data” (P13)

The majority of participants who successfully completed the VTE-PRO trial confirmed that they understood the study processes and objectives, had adequate social support from family and friends, and had no concerns regarding the heparin injection. These participants did not report any significant challenges to study participation:

“No challenges with injections, participation, social influences. It didn’t hurt and it was very simple to do” (P3)

“I have been able to take the injection pretty much plus or minus ½ hour each day and I haven’t missed any days, and it hasn’t been uncomfortable … I think [the trial] has been fairly straightforward. I understood what I was to do and it went well” (P5)

Participants who did not have one of these proposed IRIS-AR supports in place cited corresponding challenges pertaining to trial knowledge, study responsibilities, and an inability to complete the injections.

### Barriers to trial accrual and retention

The primary reasons for nonparticipation included a fear of self-administering the heparin injections, a lack of understanding of the study processes and objectives, and a fear of experiencing side effects caused by the injection while far from a hospital, as shown in the following participant quotes:

“I just don’t like needles … just the thought” (P15)

“When I found out I would have to … administer my own needle I pulled back … My knowledge of myself not being qualified or trained to administer a needle … I could really mess it up with an air ball, and when I had a reaction there would be nobody here to help me” (P12)

“It wasn’t so much to do with the injection, it was more the drug … I live 40 minutes outside of Hamilton, in the country” (P14)

### Perceptions of IRIS-AR acceptability

Following barrier and facilitator assessment, participants were asked to evaluate the acceptability of the proposed IRIS-AR intervention. Feedback regarding each intervention component is presented below.

#### Inform

The first component of IRIS-AR was use of information booklets and counseling. Most participants supported the use of information booklets, and perceived them as low-stake interventions that might promote retention. As one participant stated:

“I’m a great one for research and a book … that would have made a difference” (P12)

Participants highlighted the need to include information regarding injection safety in the information booklet, citing fear of needles as the primary barrier to nonparticipation, as demonstrated in the following quote:

“I think you should reiterate [in the information booklet] that it [the injection] doesn’t really hurt. And the needle is really small, and you can’t kill yourself!” (P9)

Participants supported the use of the ‘inform’ counseling session as a means to tailor the IRIS intervention components to each trial participant. However, they suggested that these sessions might be more effective if they took place after, rather than before, their surgery (which was the standard practice at the time), as seen below:

“I just didn’t understand the whole process, like I understood the surgery but you don’t know what you are in for or what to expect, and I think I would have understood [the trial] better after [surgery]. You are just so scared [of the actual surgery] that you just don’t think it out [trial participation].” (P16)

“I really have to come back to the timing issue. The suggestion would be, you know, after the surgery when they were still in hospital, might have been a better time to [recruit].” (P18)

#### Remind

The second component of the IRIS-AR, ‘remind’, was the use of daily reminders to administer heparin injections. Participants were generally in favor of this intervention, but were divided on the optimal frequency of the reminders. Those in favour of the daily reminders believed they would reduce rates of noncompliance and would support trial participants, as demonstrated in the following participant quote:

“Well every day [reminders] are good for the reason that you get to know how that person feels everyday, and plus, there could be an issue one day, so everyday I think is a good idea, especially after an operation … yeah, you need an everyday reminder” (P6)

Others strongly disagreed with the notion of being contacted daily by the study team for 30 days, as seen below:

“That would have my back up in a minute … It would feel like having a nagging mother around again … expecting that phone to ring everyday with the same, even if it is a taped, reminder, would have made me feel very fettered. I understand human nature and we probably do put off things that we don’t relish, like poking ourselves with needles.” (P12)

“The daily pep talk, or reminder, would probably be helpful for some people … but I don’t know, I think myself, if you did that to me every day for 30 days, I would get annoyed” (P9)

Most participants agreed that the frequency and modality of reminders should be tailored to the individual, ideally during the ‘inform’ counseling session, as demonstrated in the following quotes:

“I suppose giving people the option of calling by a certain time, and then being fair game for a gentle reminder … if they had to be nudged twice then I don’t think they are really in an altruistic frame of mind anyway” (P12)

“I do think the daily reminder is too much … I think every 3 or 4 days is fine” (P1)

#### Involve

The third component of IRIS-AR, ‘involve’, aimed to include family and friends (if consent was provided by the trial participant) to support the participant during the trial period. Most participants who completed the trial already had the support of family and friends, and agreed that this support was important, as seen in the following quotes:

“Absolutely. They would know what it [the trial] is all about. I think that it is a good idea to have them [family] involved” (P2)

“It is useful to do it with the family member present. Because people at that stage are … the patient is a little bit out of it. So, it might be a good idea to have somebody that is a little more clearheaded in there at the same time” (P17)

Participants highlighted that the principal roles of family and friends was to provide reminders, and assist with needle injections, as demonstrated below:

“I think probably a friend or family would be a good idea. I mean, I am a little different in that sense where I’m independent and I can give myself the injections … but some people [it might help], especially if they do not feel too comfortable giving themselves a needle” (P6)

Only one participant dissented to involving family, and preferred to be independent, as demonstrated below:

“No need to involve family. I just don’t need their constant … in fact, it bothers me when they are constantly on me!” (P7)

Finally, in contrast to our initial survey findings, participants highlighted that family concerns regarding trial participation would not have dissuaded them from consenting to the trial. Rather, participants strongly reiterated that the decision to participate in the trial was theirs, and was not affected by social influences.

#### Support

The final proposed component of IRIS-AR, ‘support’, leveraged the ICC program to support participants with needle injections. As we hypothesized, the majority of participants highlighted that they did not require support with the injections, yet believed the support intervention might improve accrual and retention barriers for those afraid of needles, as seen below:

“I would say the first time for me [to self-administer the needle], it was okay, but somebody else, might be kind of reluctant on giving themselves needles, so they might need [support] a couple of times” (P1)

“Knowing somebody was there if I needed them would be helpful. In my case, I didn’t need them, but somebody might need them” (P3)

This component of IRIS-AR was particularly favored by participants who did not partake in the IRIS-AR trial, suggesting that it may have improved accrual, as seen below:

“If I had somebody to do it [the injection] for me, then yeah, I would do it [participate] that way. If someone could come in and give me the needle and not disturb my lifestyle” (P11)

### Summary of study results

Most participants who completed the VTE-PRO did not experience significant challenges with the study processes. These individuals typically did not have fears or challenges pertaining to needle injection and had the support of family or friends. Participants who did not participate in the VTE-PRO trial most commonly cited fears pertaining to needle injections. Participants generally supported the implementation of the IRIS-AR intervention as a support strategy for the VTE-PRO trial and perceived the use of counseling sessions, information booklets and the involvement of family as low-stake interventions that could be implemented to improve accrual and retention. Participants were most divided over the acceptability of the daily reminders to administer the heparin injections, suggesting the modality and frequency of reminders should be tailored to the individual participant at the time of the ‘inform’ counseling session. Finally, most participants highlighted that they would not require the support of the nursing ICC program to administer injections. Participants who did fear needle injections highlighted that the support of an ICC nurse may have eased their worry; however, some did not feel that the added support of a nurse would have changed their decision not to participate.

## Discussion

The IRIS-AR support strategy was designed to improve rates of accrual and retention the VTE-PRO trial, which aims to reduce venous thromboembolic events for patients undergoing lung resection. The final IRIS-AR support strategy is comprised of information booklets and counseling sessions to identify unique participant challenges to participation and address trial concerns (‘inform’), reminders to administer injections (‘remind’), involvement of family/caregivers in study processes (‘involve’), and nursing support for individuals unable to self-administer the injections (‘support’) [29].

The importance of considering the impact of participant behaviour on the decision to participate and remain in clinical trials has been increasingly recognized [30, 31]. A recent article reported that 19% of registered clinical trials were terminated early due to low accrual rates [32]. Poor accrual and retention often lead to the premature closure of trials and pose a significant threat to the validity of trial results [33]. Reported rates of study termination due to inadequate accrual or retention are even higher in cancer trials at approximately 28% [34]. Involving the target population in the design of an intervention, a process known as integrated knowledge translation, may facilitate more acceptable interventions and subsequently enhance the target behavior [15]. The involvement of the target population in this study revealed a number of issues not previously considered by the study team. For instance, we perceived family members as key influencers of trial participation, but participants strongly believed that the decision to participate in the trial was their own and was not affected by their family members. Similarly, participants provided insight on optimized timing of VTE-PRO trial recruitment and consent, which they believed would improve accrual rates. Rather than having the study presented preoperatively (which is standard practice for many surgical studies), patients preferred to be approached postoperatively. Preoccupied with their upcoming procedure, participants reported feeling less equipped to think through the study requirements, and would have been more inclined to participate postoperatively once they had been assured of the procedure success.

In addition to involving the target population in the design and implementation of intervention components, we recommend the use of a theoretical framework. Despite a plethora of literature to describe barriers and facilitators to trial accrual and retention, there are only few studies that have used a theoretical framework to understand these factors in the context of a randomized trial [30]. To the best of our knowledge, this study is the first to utilize a theoretical framework to develop a support strategy to improve rates of RCT accrual and retention for a surgical population. We present this work as an example of how theory can be used efficiently to facilitate recruitment and adherence to clinical trials, particularly those using patient-directed interventions.

One challenge to using theory is the significant time and resources required to comprehensively identify factors driving behavior [25]. We consulted with KT experts to determine if there was an abbreviated version of the TDF that could be used in this context, but were unable to identify such tools. In this study, we present a modified method of using the TDF for intervention design within the constraints of a randomized trial. First, we recommend study coordinators routinely track reasons for study nonparticipation and attrition in detail. Such tracking allowed us to quickly focus on the TDF domains that were likely driving low rates of accrual and retention. This was an efficient contrast to traditional uses of the TDF, which require significant time and resources. Second, we reiterate the importance of involving the target population to ensure the accuracy of identified barriers and acceptability of proposed interventions prior to implementation. Our key informants confirmed the accuracy of the TDF domains we mapped using survey data, but provided further context that we were unable to glean from survey data alone. We propose this method as a potential mechanism of using theory to enhance compliance to study interventions, without imposing significant delays and resource requirements on the existing trial [25].

We posit that the IRIS-AR intervention components can be generalized to other trials involving patient-directed interventions, given their consistency with other evidence on successful trial recruitment strategies [34, 35]. We also believe there is strength in the multipronged intervention approach of IRIS-AR, given the little evidence to suggest that a single strategy is significantly associated with increased rates of accrual and retention [36, 37]. Rather, a multipronged intervention is more likely to address the needs of a broader study population [36, 37].

Finally, we encourage the leverage of existing programs to facilitate trial accrual and retention. In this study, we harnessed the existing nurse-led ICC program to serve a subset of the study population that were uncomfortable with administering injections themselves. Key informant interviews revealed that while most participants approved of the ‘support’ intervention which integrates the SJHH ICC program, only few reported that they would have required this support. This is an important finding, as it suggests the ‘support’ component would likely only be used by a small proportion of study participants and would not impose significant time and resource implications to the ICC program.

Finally, this study is not without limitations. First, patients who successfully completed the IRIS-AR study typically did not experience any major barriers to implementation and those who did highlighted only 1 or 2 major barriers to participation or adherence. Therefore, it is likely that not all patients will require all components of the IRIS intervention, and that some modifications specific to each patient might be warranted. Second, participants who feared needle injection did not believe the IRIS components would have changed their decision to participate in the trial. This suggests that IRIS-AR, in the context of the VTE-PRO trial, may improve rates of retention and satisfaction, but may not improve rates of VTE-PRO accrual. Similarly, we suggest that certain challenges may be unique to accrual versus retention, or vice versa. Modifications to the IRIS intervention may focus specifically on improving accrual or retention, depending on the unique challenges facing study teams. We will aim to pilot the proposed IRIS-AR strategy to evaluate whether rates of VTE-PRO accrual and retention improved following implementation, and whether additional interventions are warranted. Finally, we present a pragmatic mechanism to integrating theory to design interventions for improved trial participation. However, this abbreviated model is certainly not comprehensive, and potential theoretically rooted drivers may have been overlooked. As such, we strongly recommend involving the target population throughout the support intervention design and implementation process to minimize such risks. In keeping with recommended KT models, iterative evaluation and tailoring of the support interventions throughout the trial period is also prudent [15, 20].

In summary, the IRIS-AR presents a novel approach to trial engagement and leverages existing support to promote trial participation and retention. We hypothesize that IRIS-AR will be feasible and cost-effective to implement and will result in a more representative study sample of the overall thoracic population. The methodology used in this study can be replicated to inform the development of support strategies for other randomized trials.

## Conclusions

Principles of implementation science should be routinely adopted to support patient-directed interventions that are tested in clinical trials. Use of theory and integrated KT to design support strategies for intervention adoption and compliance may improve study accrual and retention and enhance the pragmatism and generalizability of RCT results. The IRIS-AR presents a patient-centered intervention that uses such principles and leverages existing programs to facilitate engagement with the VTE-PRO trial. The proposed strategy can likely be adapted to improve compliance with other patient-directed interventions.

## Data Availability

The data generated and/or analyzed during the current study are available from the corresponding author on reasonable request.

## Abbreviations

COM-B: Capabilities, opportunities and behaviour
ICC: Integrated Comprehensive Care
IRIS-AR: Inform, Remind, Involve and Support to improve Accrual and Retention
KT: Knowledge translation
RCT: Randomized controlled trial
SJHH: St. Joseph’s Healthcare Hamilton
TDF: Theoretical domains framework
VTE: Venous thromboembolism
VTE-PRO: Venous Thromboembolism Prophylaxis
